# Performance Assessment of a Junctionless Heterostructure Tunnel FET Biosensor Using Dual Material Gate

**DOI:** 10.3390/mi14040805

**Published:** 2023-03-31

**Authors:** Haiwu Xie, Hongxia Liu

**Affiliations:** 1 Key Laboratory for Wide-Band Gap Semiconductor Materials and Devices of Education, The School of Microelectronics, Xidian University, Xi’an 710071, China; 2 The School of Physics and Electronic Information Engineering, Qinghai Normal University, Xining 810016, China

**Keywords:** biosensors, band to band tunneling (BTBT), tunnel field effect transistor (TFET), dual material gate heterostructure junctionless tunnel field-effect transistor (DMG-HJLTFET)

## Abstract

Biosensors based on tunnel FET for label-free detection in which a nanogap is introduced under gate electrode to electrically sense the characteristics of biomolecules, have been studied widely in recent years. In this paper, a new type of heterostructure junctionless tunnel FET biosensor with an embedded nanogap is proposed, in which the control gate consists of two parts, namely the tunnel gate and auxiliary gate, with different work functions; and the detection sensitivity of different biomolecules can be controlled and adjusted by the two gates. Further, a polar gate is introduced above the source region, and a P+ source is formed by the charge plasma concept by selecting appropriate work functions for the polar gate. The variation of sensitivity with different control gate and polar gate work functions is explored. Neutral and charged biomolecules are considered to simulate device-level gate effects, and the influence of different dielectric constants on sensitivity is also researched. The simulation results show that the switch ratio of the proposed biosensor can reach 10^9^, the maximum current sensitivity is 6.91 × 10^2^, and the maximum sensitivity of the average subthreshold swing (SS) is 0.62.

## 1. Introduction

Biosensors based on metal oxide semiconductor field effect transistors (MOSFETs) are very popular for label-free detection due to their compactness and energy efficiency, especially the possibility of on-chip integration. The electrical detection principle behind field effect transistor (FET) biosensors is dielectric modulation, in which an electrical nanogap is created under a gate electrode. Dielectric modulated field effect transistors (DM-FETs) can detect not only charged biomolecules but also uncharged biomolecules since the effective capacitance under the gate will be influenced by the charge of biomolecules. As a result, the channel conductance in DM-FETs varies with the variation of detection targets, and the electrical variation can be further processed by measurement systems. In N-type FETs, positively charged molecules will lead to the accumulation of electrons on the sensing channel, resulting in an increase in device conductance, and negatively charged targets will decrease the electron concentration and restrain the device conductance. In P-type FETs, the conclusion is reversed [1,2]. However, MOSFET biosensors are limited by the 60-mV/Dec subthreshold swing (SS), and they have long response times in applications. Tunnel field effect transistors (TFETs), working on band to band tunneling (BTBT) mechanisms, can overcome this limitation, and the fabrication processes for them are compatible with conventional CMOS. Many researchers have reported TFET-based biosensors, which can ensure high sensitivity and low power consumption in applications for biomolecule detection [3,4]. The sensitivity of TFET-based biosensors can be evaluated by changes in electrical parameters caused by permittivity variation in charged or uncharged biomolecules, such as drain current, threshold voltage, subthreshold swing, etc. [5,6].

However, TFET-based biosensors already exist that need ultra-steep doping profiles in active regions to form abrupt junctions, leading to random dopant fluctuations (RDFs). Moreover, the quantum tunneling barrier width between the source and the channel interface in conventional TFET biosensors will significantly affect the ON-state current.

To overcome stringent demands of fabrication and avoid high thermal budgets in conventional TFETs, the junctionless tunneling field effect transistor (JLTFET) has been extensively studied in recent years [7,8,9,10]. Fabricating abrupt metallurgical junctions at high temperatures is not necessary with JLTFETs, in which uniformly high-doping concentrations are adopted in the source, channel, and drain regions. Moreover, the source and drain regions in JLTFETs are formed by the charge plasma concept, in which appropriate work functions for electrodes are chosen to further avoid a high thermal budget [11,12,13,14,15].

In this article, we propose a dual material gate heterostructure junctionless tunneling field effect transistor (DMG-HJLTFET) biosensor for the first time [16,17,18,19,20,21,22]. The proposed DMG-HJLTFET biosensor has three novel aspects. First, the control gate is divided into two parts, namely the tunnel gate and auxiliary gate, using a gate-engineered concept, in which the work functions of the tunnel gate and auxiliary gate are carefully optimized to obtain high current sensitivity and a high switch ratio. Second, an optimized InAs/GaAs_0.5_Sb_0.5_ heterostructure is adopted between the source and the channel interface, which can effectively narrow the tunneling barrier width in this region due to the properties of III-V compound semiconductor materials. Third, the charge plasma concept is used to form P^+^-I-N^+^ structures by selecting appropriate work functions for the polar gate (PG) and control gate (CG), which can avoid the formation of abrupt junctions due to uniform doping concentrations in this kind of device. Nanogaps are located below the TGs, and charged or uncharged biomolecules will lead to gate effects when they enter these gaps [23,24,25,26,27,28].

In Section 2, the basic device structure and the initial device parameters are presented, and then the essential simulation models and methods are introduced. Section 3 indicates the optimization process and simulation results of DMG-HJLTFETs. Section 4 concludes the paper.

## 2. Geometric Structure and Simulation

**Figure 1:**
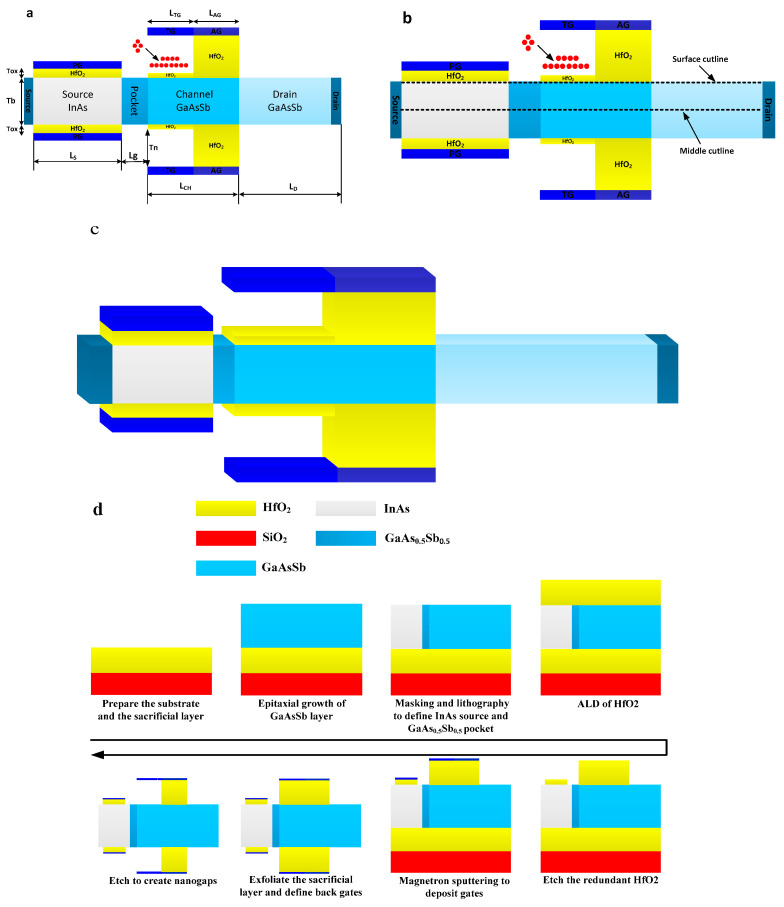
(**a**) Cross-sectional view of DMG-HJLTFET biosensor; (**b**) section diagram of cutting lines; (**c**) 3-D graphic of DMG-HJLTFET; (**d**) tentative fabrication flow of DMG-HJLTFET.

Figure 1a shows the cross-sectional view of the DMG-HJLTFET biosensor. As can be seen from Figure 1a, a GaAs_0.5_Sb_0.5_ (0.5 is the optimized value, which we analyze later) pocket structure is introduced between the source and the channel, which can effectively enhance the tunneling rate in this region [20]. The control gate electrode consists of a TG and an AG using two different metals. The ON-state current and the OFF-state current of the DMG-HJLTFET biosensor can be optimized by choosing appropriate work functions for the TG and AG. HfO_2_ as a dielectric material under the TG is etched out to form a nanogap for biosensing purposes. The charge plasma concept is used to form the source region, which is controlled by the PG. All the regions adopt uniform doping concentrations.

The length of the proposed device is 67 nm, and detailed parameters are listed in Table 1.

We simulate the proposed device in ATLAS Silvaco TCAD software, version 5.20.2.R. The nonlocal BTBT model (BBT.NONLOCAL) is activated to consider forward and reverse tunneling currents. To include the effects caused by high doping and thinner oxide, Hansch’s quantum confinement model (HANSCHQM) is activated. We also activate the Schenk model for tunneling (SCHENK.TUN), the Fermi statistics (FERMI) model, and the band gap narrowing (BGN) model, and we refine the simulation mesh and select the simulation method with better convergence.

It is worth emphasizing that a dual material gate structure can be realized by the molecular beam epitaxy (MBE) method, using the method in article [25]. Moreover, a III-V compound structure can be fabricated by the metal organic chemical vapor deposition (MOCVD) method [27,28,29]. Figure 1c shows a 3-D graphic of the DMG-HJLTFET, and Figure 1d shows the tentative fabrication flow for the DMG-HJLTFET.

**Figure 2:**
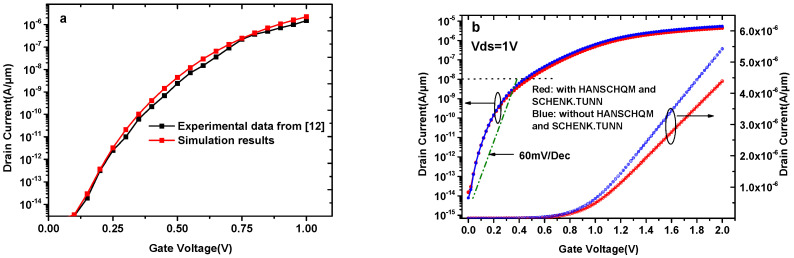
(**a**) Simulation models calibrated by reproducing the results of [12]. (**b**) Transfer characteristics of DMG-HJLTFET biosensor with and without HANSCHQM and SCHENK.TUN.

The presence of biomolecules will alter the dielectric constant of the nanogaps, and these nanogaps in our simulation are occupied by insulating material with the same dielectric constant as the corresponding biomolecule. In addition, the charges of biomolecules are considered to be interface states between HfO_2_ and the equivalent material. Although no experimental results are available for this kind of TFET biosensor, based on experimental data and theoretical calculations, the dielectric modulated double gate tunnel field effect transistor (DG-TFET) biomolecule sensor in reference [12] is well calibrated. Therefore, the BTBT model in this article is calibrated by reproducing the results reported in [12], as shown in Figure 2a.

Figure 2b shows the transfer characteristics of the DMG-HJLTFET biosensor with and without HANSCHQM and SCHENK.TUN. In this condition, nanogaps are occupied by air (k = 1). To consider trap-assisted tunneling, the SCHENK.TUN model is activated in our simulation. This model gives the field-effect enhancement factors as an analytic function, and Atlas works out this function through several intermediate quantities. Moreover, it is necessary to consider the effects near the oxide interface because the minimum oxide layer thickness in our simulation is as small as a few nanometers, and HANSCHQM is suitable for accurate simulations of quantum mechanical confinement effects near the gate oxide interface. As can be clearly seen from this figure, the two models have a significant effect on both OFF-state current (Vgs = 0 V) and ON-state current (Vgs = 2 V). The maximum ON-state current with HANSCHQM and SCHENK.TUN is 4.39 × 10^−6^ A/μm, and the switch ratio can reach 10^9^, while the average SS is 82mV/Dec.

**Figure 3:**
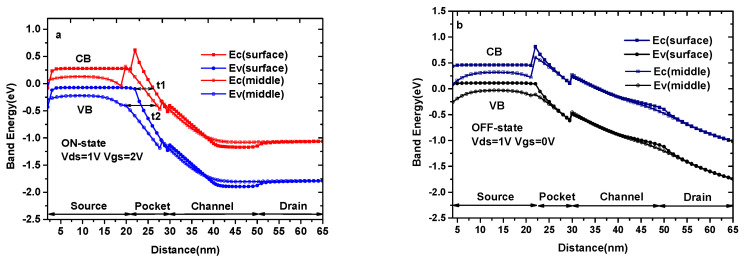
(**a**) The ON-state energy band diagram of DMG-HJLTFET; (**b**) The OFF-state energy band diagram of DMG-HJLTFET.

Figure 3a and b show the ON-state and OFF-state energy band diagram of DMG-HJLTFET (k = 1), respectively, which explain the reason for maintaining ^a^ high ON-state current and switch ratio. It is found that the valance band and conduction band between the source and channel are very close to each other, ensuring a smaller tunneling distance at the surface region, as shown by t1. At the same time, tunneling distance at the middle region, represented by t2, is not very large, so the middle region also contributes a small amount to the tunneling current. The tunneling width and effective tunneling area at the surface and middle region in this biosensor are distinctly improved by InAs/GaAs_0.5_Sb_0.5_ heterostructures. Moreover, the TG with a work function of Φ_M1_ can lower the minimum value of the conduction band at the pocket region, which can further improve the tunnel process in this region; the AG with a work function of Φ_M2_ can produce an extra barrier in the channel region, which can help to improve the OFF-state current and switch ratio. In Figure 3b, we can see that the tunneling distance of the conduction band and valance band at the source/channel interface in both regions is too large to tunnel effectively.

Obviously, the current capacity of the DMG-HJLTFET will be affected by the composition variation in GaAs_y_Sb_1-y_, and GaAs_0.5_Sb_0.5_ is an optimized value.

**Figure 4:**
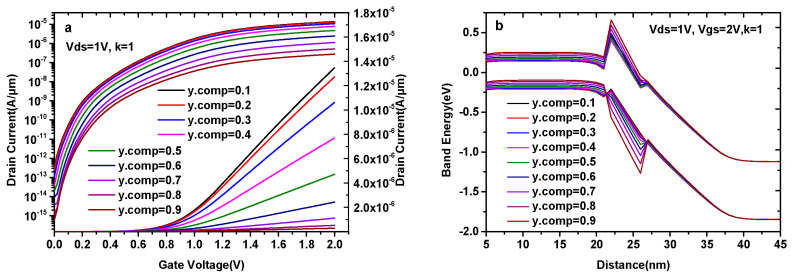
(**a**) Transfer characteristics of DMG-HJLTFET and (**b**) energy band diagram of DMG-HJLTFET with different y in GaAs_y_Sb_1−y_.

In Figure 4, we depict the transfer characteristics and energy band diagram of a DMG-HJLTFET with a different y. As can be seen in Figure 4a, both the ON-state current (Vgs = 2.0 V) and the OFF-state current (Vgs = 0 V) decrease with the increase in y, and the OFF-state current is close to 10^−14^ A/μm until y.comp = 0.5. For the sake of compromise, we choose y.comp = 0.5. Figure 4b shows the ON-state energy band diagram of the DMG-HJLTFET with a different y. It is found that the value of the conduction band in the GaAs_y_Sb_1−y_ region increases with the increase in y, while the valance band changes in the opposite direction, indicating a reduction in the effective tunneling area at the source/pocket interface. The OFF-state energy band diagram of the DMG-HJLTFET with a different y is similar to that of the ON-state diagram. However, there are still significant electrons that can tunnel from the source to the pocket due to the small tunneling distance when y.comp<0.5. Therefore, the variation of energy bands leads to the change in current in Figure 4a.

## 3. Results and Discussion

### 3.1. Influence of Tunnel Gate Work Function

Formula (1) gives the current sensitivity for biosensors:

(1) $$I_{Sensitivity}=\frac{I_{D,K}}{I_{D,air}}$$

In this paper, we continue to use this definition. I_D,k_ and I_D,air_ represent the drain current of the DMG-HJLTFET biosensor with biomolecules and without biomolecules, respectively.

**Figure 5:**
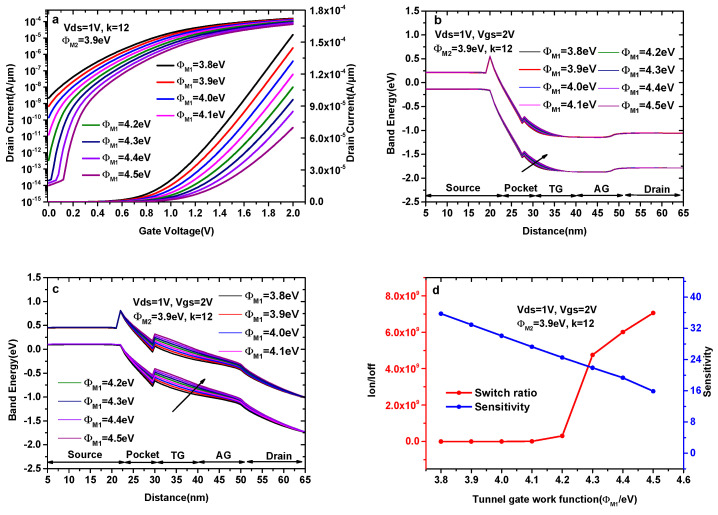
(**a**) Transfer characteristics of DMG-HJLTFET; (**b**) the variation of the ON-state energy band diagram; (**c**) the variation of the OFF-state energy band diagram; and (**d**) sensitivity and switch ratios (k = 12) with different tunnel gate work functions (Φ_M1_).

Figure 5a–c indicate the transfer characteristics and the energy band diagrams of the DMG-HJLTFET with different tunnel gate work functions (Φ_M1_), wherein we keep k = 12, Φ_M2_ = 3.9eV and Φ_PG_ = 5.9 eV. As shown in Figure 5a, the selection of Φ_M1_ is crucial for obtaining a higher Ion/Ioff and a lower average SS. Although the ON-state current gradually decreases with the increase in Φ_M1_, the switch ratio and SS value are very poor when Φ_M1_<4.3 eV.

In Figure 5b, it is observed that the tunneling effective area decreases with the increase in Φ_M1_, consistent with the variation of the maximum ON-state current in Figure 5a. The mechanism of OFF-state current variation can be explained by the OFF-state energy band diagram in Figure 5c. There is overlap between the source valance band and the pocket conduction band before Φ_M1_ = 4.3 eV; i.e., the tunneling process still exists when Φ_M1_<4.3 eV, resulting in high Ioff in these conditions, while the overlap disappears after Φ_M1_ = 4.3 eV, causing the OFF-state current to become very small, as shown in Figure 5a.

Figure 5d indicates the variations in sensitivity (k = 12) and switch ratio with different tunnel gate work functions. The surface potential and electric field under the tunnel gate decrease with the increase in Φ_M1_, leading to the elevation of the conduction band and valance band in the pocket region, and this trend becomes more obvious with the increase in Φ_M1_. As a result, the tunneling effective area decreases with the increase in Φ_M1_; in other words, I_D,k_ decreases with the increase in Φ_M1_. Therefore, the sensitivity decreases with the increase in Φ_M1_. As can be seen in Figure 5d, the sensitivity of the DMG-HJLTFET decreases from 35.76 to 15.89 when Φ_M1_ increases from 3.8 eV to 4.5 eV, whereas the switch ratio is greater than 10^9^ after Φ_M1_ = 4.3 eV. Therefore, the optimal work function value for the TG is chosen as 4.3 eV. To gain insight into the design and optimization process of the TG, the detailed variation of the sensitivity and switch ratio with different Φ_M1_ is shown in Table 2.

### 3.2. Influence of Auxiliary Gate Work Function and Polar Gate Work Function

In this section, we discuss the influence of Φ_M2_ and Φ_PG_ on electrical performance.

**Figure 6:**
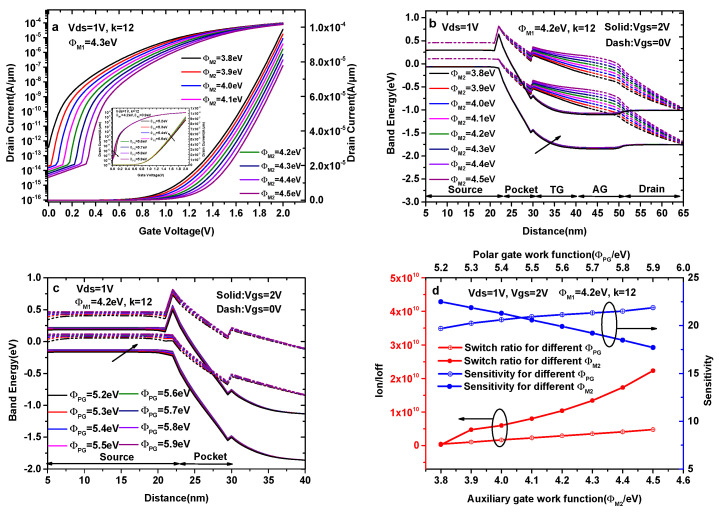
(**a**) Transfer characteristics of DMG-HJLTFET with different auxiliary gate work functions (Φ_M2_); (**b**) energy band diagram of DMG-HJLTFET with different auxiliary gate work functions (Φ_M2_); (**c**) energy band diagram of DMG-HJLTFET with different polar gate work functions (Φ_PG_); (**d**) the variation of sensitivity and switch ratios with different Φ_M2_ and Φ_PG_.

Figure 6a shows the transfer characteristics of a DMG-HJLTFET with different values of Φ_M2_, where Φ_M2_ increases from 3.8 eV to 4.5 eV in steps of 0.1 eV. In Figure 6a, it is found that the current at Vgs = 2.0 V decreases with the increase in Φ_M2_. However, the OFF-state current of Φ_M2_ = 3.8 eV is 3.79 × 10^−13^ A/μm, which is one or two orders of magnitude higher than the OFF-state current in other cases. Figure 6b shows the ON-state and the OFF-state energy band diagram of a DMG-HJLTFET with a different auxiliary gate work function (Φ_M2_), where it is observed that the valley of conduction band in the pocket region increases with the increase in Φ_M2_ at Vgs = 2.0 V and Vgs = 0 V. As a result, the ON-state effective tunneling area at the source/pocket interface decreases with the increase in Φ_M2_, and the OFF-state tunneling distance between the source valance band and the pocket conduction band increases with the increase in Φ_M2_. Moreover, an overlap between the source valance band and the pocket conduction band exists at Φ_M2_ = 3.8 eV in the OFF-state. These variations in the energy band are highly consistent with the current change in Figure 6a. Therefore, Φ_M2_ = 3.9 eV is chosen as the optimal value for the auxiliary gate.

The inset indicates the transfer characteristics of a DMG-HJLTFET with different values of Φ_PG_, in which we keep Φ_M1_ = 4.3 eV and Φ_M2_ = 3.9 eV. The inset picture shows that the ON-state current at Vgs = 2.0 V gradually increases when Φ_PG_ varies from 5.2 eV to 5.9 eV in steps of 0.1 eV, whereas the OFF-state current is not obviously influenced by Φ_PG_, which remains on the order of 10^−14^ A/μm. The reason for this outcome is that the polarization charge forming in the source region is deeply influenced by Φ_PG_, and the number of polarization charges increases with the increase in Φ_PG_. Figure 6c illustrates the energy band variation of DMG-HJLTFET with different Φ_PG_; although the energy band gap among different Φ_PG_ is not obvious at Vgs = 2.0 V, the position of the conduction band and valance band in the source region becomes increasingly higher when Φ_PG_ increases, resulting in the increase in the effective tunneling area at the source/pocket interface.

Additionally, the position of the conduction band and valance band in the source and pocket regions at Vgs = 0 V becomes increasingly higher, ensuring that the OFF-state current decreases with the increase in Φ_PG_. Because of the ON-state current and OFF-state current, we choose Φ_PG_ = 5.9 eV.

To better understand the influence of the AG and PG, the detailed variation of sensitivity and switch ratios with different Φ_M2_ are shown in Table 3, and Table 4 shows the variation in sensitivity and switch ratios with different Φ_PG_.

Figure 6d shows the variation in sensitivity and switch ratios (k = 12) with different auxiliary gates and polar gates. The sensitivity decreases from 22.49 to 17.71 when Φ_M2_ increases from 3.8 eV to 4.5 eV, whereas the switch ratio of Φ_M2_ = 3.8 eV is only 2.61 × 10^8^. Therefore, the AG work function is chosen as 3.9 eV to account for the ON-state current, switch ratio and sensitivity. The sensitivity increases from 19.70 to 21.87 when Φ_PG_ increases from 5.2 eV to 5.9 eV, and the switch ratio increases from 4.42 × 10^8^ to 4.76 × 10^9^. Therefore, we choose Φ_PG_ = 5.9 eV.

### 3.3. Influence of Charge Density and Dielectric Constant

**Figure 7:**
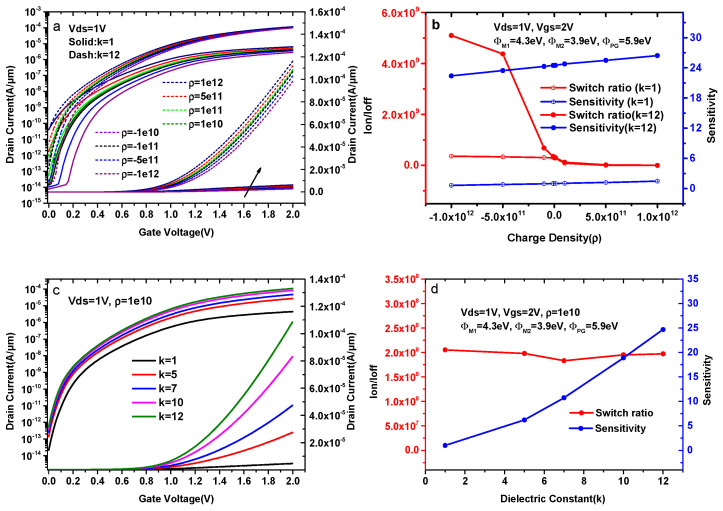
(**a**) Transfer characteristics of DMG-HJLTFET for different charge densities with a fixed dielectric (k = 1 and k = 12); (**b**) the variation of the sensitivity and switch ratio for different charge densities with a fixed dielectric (k = 1 and k = 12); (**c**) Transfer characteristics of DMG-HJLTFET for k = 1, 5, 7, 10, and 12 at a fixed charge (ρ = 1 × 10^10^ cm^−2^); (**d**) the variation of the sensitivity and switch ratio for k = 1, 5, 7, 10, and 12 at a fixed charge (ρ = 1 × 10^10^ cm^−2^).

The comparison of transfer characteristics with different charge densities is shown in Figure 7a. Figure 7a presents the plots for the positive charge and negative charge at a fixed dielectric constant (k = 1 and k = 12). The ON-sate current of k = 1 is much smaller than that of k = 12 for a certain charge density when ρ increases from −1 × 10^12^ cm^−2^ to 1 × 10^12^ cm^−2^. For ρ = 1 × 10^12^ cm^−2^, the maximum ON-state current rises by a factor of 18.1. The reason for this change is that a higher dielectric constant helps to form a p-type source and an intrinsic channel under uniform doping conditions, resulting more electrons tunneling from the source to the pocket due to an increase in surface potential. Figure 7b shows the variation of sensitivity and switch ratios with different charge densities for k = 1 and k = 12. The sensitivity of k = 1 rises by a factor of 2.28, whereas the switch ratio is reduced by three orders of magnitude when ρ increases from −1 × 10^12^ cm^−2^ to 1 × 10^12^ cm^−2^. The switch ratio of k = 12 is also reduced by three orders of magnitude, whereas the sensitivity only rises by a factor of 1.18 when ρ increases from −1 × 10^12^ cm^−2^ to 1 × 10^12^ cm^−2^.

We know that the voltage balance equation of a metal-oxide-semiconductor structure is:

(2) $$Vgs=\Psi_{s}+\Phi_{{}_{MS}}-\frac{q(\pm \rho )}{C_{eff}}$$

where ψ_S_ is the surface electrostatic potential, Φ_MS_ is the work function difference between the metal and the semiconductor, q is the value of the unit charge, ρ is the number of charged biomolecules per unit area, and Ceff is the resultant capacitance per unit area.

In our simulation, the third term of Formula (2) goes from large to small when ρ increases from −1 × 10^12^ cm^−2^ to 1 × 10^12^ cm^−2^, indicating that the surface electrostatic potential (ψ_S_) increases with the increase in ρ. Therefore, the tunneling probability near the tunnel gate increases when ρ increases from −1 × 10^12^ cm^−2^ to 1 × 10^12^ cm^−2^, resulting in an increase in the drain current and sensitivity in this device. In addition, an increase in ρ will enhance the OFF-state current, and the switch ratio decreases with the increase in ρ. In summary, the sensitivity increases and the switch ratio decreases when ρ varies from −1 × 10^12^ cm^−2^ to 1 × 10^12^ cm^−2^, and the change of sensitivity at a low k is more obvious than that of a high k.

Like the previous section, similar plots for different dielectric constant of biomolecules are presented in Figure 7c at a fixed charge (ρ = 1 × 10^10^ cm^−2^). As shown in Figure 7c, both the ON-state current and the OFF-state current increase with the increase in k. The reason for these changes is that equivalent capacitance under the tunnel gate reduces with the increase in the dielectric constant of the biomolecules. Gate effects caused by biomolecules at a fixed charge density become increasingly obvious when k goes from 1 to 12.

As reflected in the transfer characteristics, the ON-state current increases obviously with k, and the maximum ON-state current for k = 12 is 1.1 × 10^−4^ A/μm, whereas the maximum ON-state current for k = 1 is only 4.43 × 10^−6^ A/μm. The change of the OFF-state current is smaller than that of the ON-state current, and the OFF-state currents for k = 1 and k = 12 are 2.16 × 10^−14^ A/μm and 5.49 × 10^−13^ A/μm, respectively.

Combined with the changes in current capacity, Figure 7d shows the variation of the sensitivity and switch ratios for k = 1, 5, 7, 10, and 12 at ρ = 1 × 10^10^ cm^−2^. It is clear that the sensitivity of DMG-HJLTFET biosensor increases from 1.01 to 24.7 when k goes from 1 to 12, and the switch ratio of DMG-HJLTFET biosensor is always on the order of 10^8^.

In fact, compounds having the ability to cause biological interactions possess high dielectric constant, such as acetylene tetrabromide, which causes acute intoxication in a human being if exposed unconditionally and has a dielectric constant of 7; pyridine, which is used widely in agrochemicals; and denatured alcohol, which has a dielectric constant of 13 [23]. Next, we use these two substances to analyze the linearity and selectivity of the proposed device.

**Figure 8:**
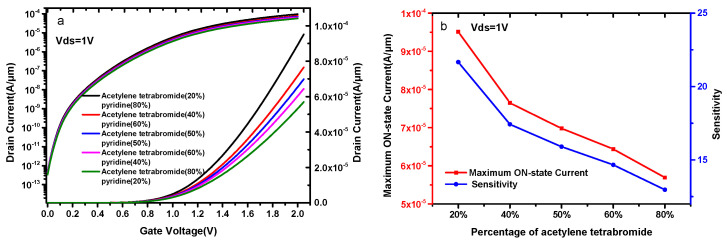
(**a**) Transfer characteristics of DMG-HJLTFET for different contents of acetylene tetrabromide and pyridine; (**b**) the variation of the maximum ON-state current and sensitivity for different contents of acetylene tetrabromide and pyridine.

Figure 8a compares the current capacity of a DMG-HJLTFET corresponding to different contents of acetylene tetrabromide and pyridine. It is found that the DMG-HJLTFET biosensor shows different selectivity for acetylene tetrabromide and pyridine. The current capacity of the DMG-HJLTFET decreases with the increase in acetylene tetrabromide percentage. The maximum ON-state current is 9.51 × 10^−5^ A/μm when the acetylene tetrabromide percentage is 20%, and the maximum ON-state current drops to 5.69 × 10^−5^ A/μm when the acetylene tetrabromide percentage increases to 80%. The DMG-HJLTFET shows higher selectivity for materials with high dielectric constants, and materials with higher dielectric constants are easier to detect. Figure 8b indicates the sensitivity with different levels of acetylene tetrabromide and pyridine. The sensitivity is 21.7 when the acetylene tetrabromide percentage is 20%, while the sensitivity is 12.9 when the acetylene tetrabromide percentage is 80%.

The SS sensitivity of biosensor is defined as follows:

(3) $${SS}_{sensitivity}=\frac{{SS}_{air}-{SS}_{bio}}{{SS}_{air}}$$

**Figure 9:**
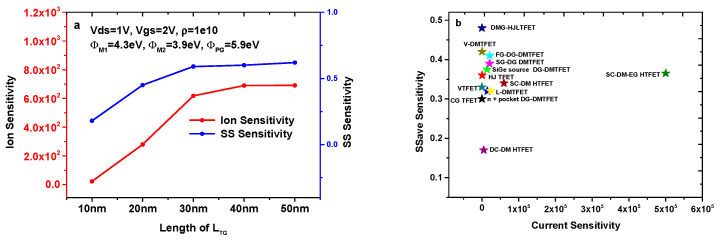
(**a**) Ion sensitivity and SS sensitivity for different L_TG_ (k = 12); (**b**) comparison of different biosensors based on TFET.

Figure 9a shows the sensitivity variation of the DMG-HJLTFET biosensor with different L_TG_. It is very clear that the current sensitivity and SS sensitivity increase with the enhancement of L_TG_. The values of current sensitivity and SS sensitivity are 6.91 × 10^2^ and 0.62, respectively, when L_TG_ = 50 nm.

Figure 9b compares the sensitivity of different TFET-based biosensors. Details of different TFET-based biosensors are listed in Table 5. In this article, for fairness of comparison, we adopt the same device thickness (10 nm) and device length (100 nm) for these different TFET-based biosensors. It is found that the DMG-HJLTFET possesses the maximum SS sensitivity due to its structural innovation. Although the SC-DM-EG HTFET has a current sensitivity of 5 × 10^5^, the SS sensitivity is clearly less than 0.4. The SS sensitivity of other structures is obviously smaller than that of the DMG-HJLTFET, and their current sensitivity is far less than 5 × 10^5^.

## 4. Conclusions

An insightful analysis for a dual material gate heterostructure junctionless tunneling field effect transistor (DMG-HJLTFET) biosensor has been presented in this work. An InAs/GaAsSb heterojunction is adopted between the source and the channel to improve the band-to-band tunneling (BTBT) rate, and a pocket structure formed by GaAs_0.5_Sb_0.5_ is inserted to further enhance the electron tunneling process at the source/channel interface. Nanogaps are introduced under the gate electrode to electrically sense the characteristics of biomolecules, and gate effects occur when biomolecules enter the detection position. To obtain higher sensitivity and a higher switch ratio, the gate electrode is divided into two parts, namely the tunnel gate and auxiliary gate; we can improve the ON-state current and the OFF-state current simultaneously by selecting appropriate work functions for the tunnel gate and auxiliary gate. The influences of different tunnel gates on sensitivity and switch ratio are explored in depth. Simulation results show that 4.3 eV is the optimal work function value for TG, and superior values for sensitivity and switch ratio can be obtained in this condition. We also study the effects of auxiliary gate work functions on device performance by keeping Φ_M1_ = 4.3 eV. Φ_M2_ = 3.9 eV is selected as the most suitable value for AG. Then, the electrical properties of positive and negative charges at k = 1 and k = 12 are researched, and the results show that the sensitivity increases, the switch ratio decreases with the increase in charge density, and the change in sensitivity at a low k is more obvious than that at a high k. Furthermore, the influence of the dielectric constant on biosensor performance is also compared, and the simulation results show that dielectrically modulated effects caused by biomolecules become increasingly obvious when k goes from 1 to 12.

## Figures and Tables

**Table 1:** The fundamental geometrical and physical parameters of the DMG-HJLTFET.

Parameter Name	Unit	Value
Length of source (L_S_)	nm	22
Length of pocket (L_g_)	nm	5
Length of channel (L_CH_)	nm	20
Length of tunnel gate (L_TG_)	nm	10
Length of auxiliary gate (L_AG_)	nm	10
Length of drain (L_D_)	nm	20
Thickness of HfO_2_ (T_OX_)	nm	2
Height of nanogap (T_n_)	nm	10
Thickness of body (T_b_)	nm	10
Work function of tunnel gate (Φ_M1_)	eV	4.3
Work function of auxiliary gate (Φ_M2_)	eV	3.9
Work function of polar gate (Φ_PG_)	eV	5.9
Uniform doping concentration (N_D_)	cm^−3^	2 × 10^18^

**Table 2:** The variation in sensitivity and switch ratios with different Φ_M1_ (Vgs = 2.0 V, Vds = 1.0 V, Φ_M2_ = 3.9 eV, Φ_PG_ = 5.9 eV).

Parameter (Φ_M1_)	3.8 eV	3.9 eV	4.0 eV	4.1 eV
Sensitivity	35.76	32.92	30.08	27.27
Ion/Ioff	7.34 × 10^4^	2.10 × 10^5^	9.48 × 10^5^	9.64 × 10^6^
Parameter (Φ_M1_)	4.2 eV	4.3 eV	4.4 eV	4.5 eV
Sensitivity	24.53	21.87	19.34	15.89
Ion/Ioff	3.07 × 10^8^	4.75 × 10^9^	6.01 × 10^9^	7.06 × 10^9^

**Table 3:** The variation of sensitivity and switch ratios with different Φ_M2_ (Vgs = 2.0 V, Vds = 1.0 V, Φ_M1_ = 4.3 eV, Φ_PG_ = 5.9 eV).

Parameter (Φ_M2_)	3.8 eV	3.9 eV	4.0 eV	4.1 eV
Sensitivity	22.50	21.87	21.28	20.59
Ion/Ioff	2.61 × 10^8^	4.75 × 10^9^	5.98 × 10^9^	8.03 × 10^9^
Parameter (Φ_M2_)	4.2 eV	4.3 eV	4.4 eV	4.5 eV
Sensitivity	19.91	19.22	18.48	17.71
Ion/Ioff	1.04 × 10^10^	1.34 × 10^10^	1.73 × 10^10^	2.23 × 10^10^

**Table 4:** The variation of the sensitivity and switch ratio with different Φ_PG_ (Vgs = 2.0 V, Vds = 1.0 V, Φ_M1_ = 4.3 eV, Φ_M2_ = 3.9 eV).

Parameter (Φ_PG_)	5.2 eV	5.3 eV	5.4 eV	5.5 eV
Sensitivity	19.70	20.25	20.61	20.94
Ion/Ioff	4.42 × 10^8^	1.05 × 10^9^	1.68 × 10^9^	2.31 × 10^9^
Parameter (Φ_PG_)	5.6 eV	5.7 eV	5.8 eV	5.9 eV
Sensitivity	21.14	21.34	21.52	21.87
Ion/Ioff	2.90 × 10^9^	3.50 × 10^9^	4.08 × 10^9^	4.75 × 10^9^

**Table 5:** Details of different TFET-based biosensors from Figure 9b.

Reference	Device name	Parameters
Reference [20]	L-DMTFET	Tsi = 10 nm, Lgap = 30 nm, Hgap = 5 nm, Vgs = 2.0 V, k = 4.
	V-DMTFET	Tsi = 10 nm, Lgap = 30 nm, Hgap = 5 nm, Lpocket = 5 nm, Vgs = 2.0 V, k = 4.
Reference [23]	DG-DMTFET	Tsi = 10 nm, Lgap = 15 nm, Hgap = 9 nm, Vgs = 2.0 V, k = 4.
	n+ pocket DG-DMTFET	Tsi = 10 nm, Lgap = 15 nm, Hgap = 9 nm, Lpocket = 5 nm, Vgs = 2.0 V, k = 4.
Reference [24]	CG TFET	Tsi = 10 nm, Lgap = 25 nm, Hgap = 10 nm, Vgs = 2.0 V, k = 4.
	HJ TFET	Tsi = 10 nm, Lgap = 25 nm, Hgap = 10 nm, Vgs = 2.0 V, k = 4.
Reference [25]	FG-DG-DMTFET	Tsi = 10 nm, Lgap = 20 nm, Hgap = 5 nm, Lgate = 60 nm, Vgs = 2.0 V, k = 4.
	SG-DG-DMTFET	Tsi = 10 nm, Lgap = 20 nm, Hgap = 5 nm, Lgate = 40 nm, Vgs = 2.0 V, k = 4.
Reference [26]	VTFET	Tsi = 10 nm, Lgap = 20 nm, Hgap = 5 nm, Lpocket = 10 nm, Vgs = 2.0 V, k = 4.
Reference [27]	DC-DM HTFET	Tsi = 10 nm, Lgap = 30 nm, Hgap = 5 nm, Lpocket = 10 nm, Vgs = 2.0 V, k = 4.
	SC-DM HTFET	Tsi = 10 nm, Lgap = 30 nm, Hgap = 5 nm, Lpocket = 10 nm, Vgs = 2.0 V, k = 4.
	SC-DM-EG HTFET	Tsi = 10 nm, Lgap = 30 nm, Hgap = 5 nm, Lpocket = 10 nm, Vgs = 2.0 V, k = 4.
This work	DMG-HJLTFET	Tsi = 10 nm, Lgap = 30 nm, Hgap = 10 nm, Vgs = 2.0 V, k = 4.
